# Adverse events of benralizumab in moderate to severe eosinophilic asthma: A meta-analysis

**DOI:** 10.1097/MD.0000000000015868

**Published:** 2019-05-31

**Authors:** Wanshu Liu, Xuesu Ma, Weikang Zhou

**Affiliations:** a Department of Dermatology, The Affiliated Hospital of Southwest Medical University, Luzhou City; b Department of Allergy, Chongqing General Hospital, Chongqing, China.

**Keywords:** adverse events, anti-interleukin-5, benralizumab, eosinophilic asthma, meta-analysis, receptor α monoclonal antibody

## Abstract

**Background:**

Benralizumab, a humanized, anti-interleukin-5 (anti-IL-5) receptor α monoclonal antibody that directly and rapidly depletes eosinophils, has shown significant efficacy in reducing asthma exacerbations and improving lung function in moderate to severe eosinophilic asthma patients. However, there is some controversy regarding the adverse events (AEs) of benralizumab and a comprehensive analysis of these AEs has not been performed. This study aimed to assess the incidence of these AEs in published randomized controlled trials (RCTs).

**Methods:**

We searched for RCTs in the Embase, PubMed and Cochrane databases that compared benralizumab with placebo in moderate to severe eosinophilic asthma patients. The outcome was the incidence of AEs during the observation period.

**Results:**

Eight RCTs were analyzed in this study. Patients treated with benralizumab had a lower risk of overall AEs (risk ratio (RR) 0.94; 95% confidence interval (CI) 0.90–0.98), serious adverse events (SAEs) (RR 0.82; 95% CI 0.68–0.98), asthma exacerbation (RR 0.72, 95% CI 0.61–0.85), bronchitis (RR 0.76, 95% CI 0.59–0.96) and sinusitis (RR 0.64, 95% CI 0.48–0.85), but had a higher risk of headache (RR 1.42, 95% CI 1.07–1.87) and pyrexia (RR 2.26, 95% CI 1.32–3.87) than patients treated with placebo. No increased incidence of death, hypersensitivity, injection-site reactions, nasopharyngitis, rhinitis, upper respiratory tract infection, influenza, cough, nausea, back pain or arthralgia was observed with benralizumab compared with placebo.

**Conclusions:**

Benralizumab reduced the risk of SAEs, asthma exacerbation, bronchitis and sinusitis, and aggravated the risk of headache and pyrexia. Other AEs were comparable between the benralizumab group and placebo group. Therefore, benralizumab is a relatively safe drug, but vigilance regarding AEs is imperative during long-term treatment.

## Abstract 2

Supplemental Digital Content is available in the text

## 1 Introduction

Asthma is a common, chronic inflammatory, respiratory disease that affects approximately 1% to 18% of individuals in different countries around the world.^[1]^ Patients with severe and uncontrollable asthma account for about 5% to 10% of total asthma patients, and require treatment with high-dose inhaled corticosteroids (ICS) and long-acting β2-agonists (LABA) to control the disease.^[2]^ However, the potential long-term side-effects of ICS and LABA cause suffering in many patients. Therefore, we urgently need more effective and safe treatments to improve the quality of life in these patients. For patients with uncontrolled severe eosinophilic asthma, the recently updated GINA guidelines recommend add-on treatment with anti- interleukin (IL)-5 receptor therapy for patients ≥12 years of age.^[1]^

IL-5 and eosinophils play key roles in the inflammatory response in T-helper 2-high asthma.^[3]^ IL-5 is the major cytokine in activating eosinophils and is pivotal for eosinophil development, maturation, and survival in tissues.^[4]^ Eosinophils release the contents of granules, causing tissue damage, promoting the progression of inflammation and are a classic feature of asthma.^[5,6]^ Benralizumab is an anti-IL-5 receptor α monoclonal antibody that can efficiently block the effects of IL-5.^[7,8]^ The published data indicate that benralizumab can completely deplete eosinophils and their (late) bone marrow progenitors by inducing antibody-dependent cell-mediated cytotoxicity executed by natural killer cells and/or macrophages both in vitro and in non-human primates in vivo.^[9]^ Numerous studies in humans have shown an adequate safety profile with a significant reduction in blood eosinophil counts following the administration of benralizumab.^[10]^

Recently, several clinical trials have shown that benralizumab has a significant role in reducing the rate of exacerbation and improving lung function in patients with moderate to severe, uncontrolled, persistent asthma.^[11–18]^ Benralizumb has also been approved by the FDA as add-on maintenance treatment for severe asthma in patients ≥12 years old with an eosinophilic phenotype. Some AEs of benralizumab during treatment in eosinophilic asthma patients are still controversial, and almost none of the available articles classify and analyze these AEs in detail.^[11–18]^ This meta-analysis is the first article to assess the risk of common AEs induced by benralizumab, and includes eight clinical RCTs involving patients with moderate to severe eosinophilic asthma who received benralizumab by subcutaneous (SC) injection^[11,12,14–18]^ or intravenous (IV) injection.^[11–13]^ The purpose of this meta-analysis was to estimate whether there are differences between the benralizumab and placebo groups in relation to common AEs to further demonstrate the safety of benralizumab.

## 2 Methods

This study followed the Preferred Reporting Items for Systematic Reviews and Meta-Analyses (PRISMA) guidelines.^[19]^

### 2.1 Search strategy

Two researchers (WSL and XSM), expert in EndNoteX8, performed systematic and independent searches of the MEDLINE, EMBASE, and the Cochrane Library databases using MeSH and free text search terms from inception to September 2018, and evaluated the title and abstract for eligibility. A combination of MESH terms such as “asthma”, “MEDI-563” and “anti-interleukin-5” and terms such as “eosinophilic asthma” and “benralizumab” were used to search. There were no limitations on study design or language, but only human and clinical studies were included in the evaluation.

### 2.2 Study selection

Studies that met the following criteria were included:

1. the studies must be placebo-controlled RCTs;
2. patients recruited into these studies were diagnosed with moderate to severe eosinophilic asthma;
3. recruited patients were ≥12 years old;
4. the intervention in these studies must include benralizumab regardless of the different drug doses; and
5. study outcomes must include AEs.

Studies that met the following criteria were excluded:

1. review articles, case reports and conference abstracts; and
2. articles where the full text was unavailable.

According to the above criteria, both reviewers independently analyzed the literature data and resolved any differences by discussion.

### 2.3 Data extraction

Both reviewers read the full text, supplementary appendix and extracted the data independently and meticulously. The following descriptive data were obtained from all included studies: first author, publication year, study type, patient characteristics, methods, duration, interventions, and follow-up time. The reviewers checked the accuracy of data extraction and resolved any differences by discussion. E-mails were sent to the authors if key data were missing.

### 2.4 Assessment of risk of bias

The reviewers completed an assessment of the risk of bias independently. This risk of bias assessment included: random sequence generation, allocation concealment, blinding of participants and personnel, blinding of outcome assessment, incomplete outcome data, selective reporting and other bias methods. Bias judgment was also carried out (low risk, unclear risk, high risk) according to the Cochrane Collaboration's tool for assessing the risk of bias.^[20]^

### 2.5 Data analysis

We calculated risk ratios (RRs) and 95% confidence interval (CI) for all dichotomous outcomes. We assessed heterogeneity between studies using the Chi-square (χ^2^) test and I^2^ statistics. A χ^2^ value < 0.1 and an I^2^ value of 50% or more was considered to represent significant heterogeneity. The heterogeneity between studies was assessed with the I^2^ statistic by calculating the percentage of total variation in the trials. When I^2^ was < 0.5 the results of a fixed effect analysis are presented; otherwise, the results of a random effect analysis are presented. A subgroup analysis of the dose of benralizumab or placebo was performed. A post hoc sensitivity analysis was performed by changing the effect model from a random effect model to a fixed effect model. The overall effects were calculated by combining the Z value with *P* < .05 to indicate statistical significance. When there were at least 10 trials for an outcome, a funnel plot was constructed to evaluate publication bias. All data were analyzed with the Review Manager (RevMan) software version 5.3. (The Nordic Cochrane Centre, The Cochrane Collaboration, Copenhagen, Denmark). The descriptive statistics were calculated using Microsoft Excel 2010 (Microsoft Corporation, Redmond, WA).

### 2.6 Definition of adverse effects

‘Adverse effects’ refer to any adverse medical events other than the treatment purpose that occurs after a therapeutic dose of the drug is administered, including events caused by or completely unrelated to the product.

### 2.7 Ethics

All the analyses were based on previous published studies; therefore, ethical approval is not necessary for systematic review and meta-analysis.

## 3 Results

### 3.1 Search results

**Figure 1:**
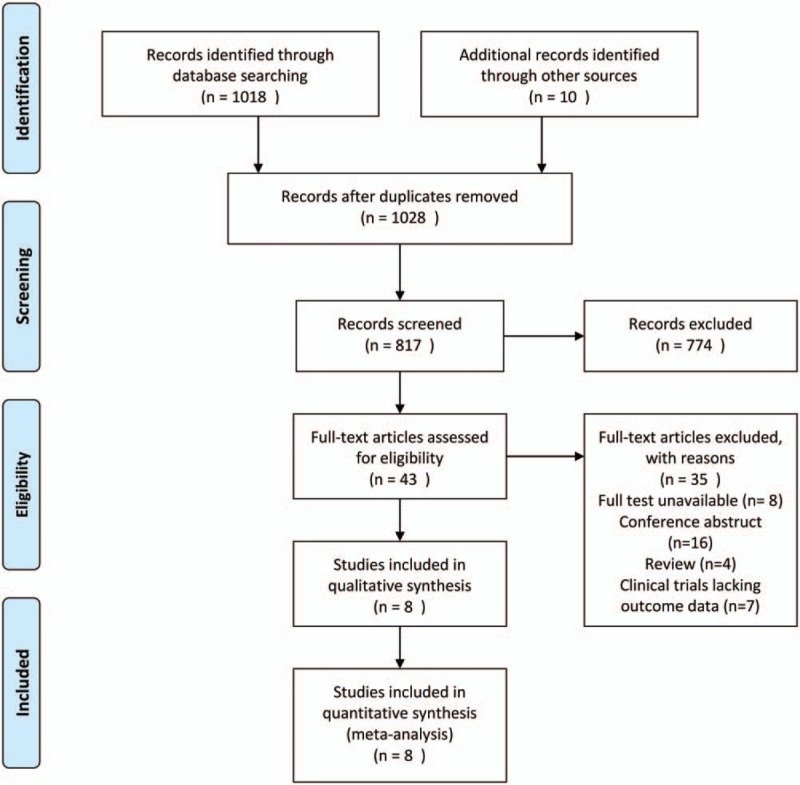
Flow diagram of study selection.

The reviewers searched a total of 1028 articles in PubMed (n = 166), Cochrane (n = 98), EMBASE (n = 754), and manual searches (n = 10). After identification of duplicates and screening of abstracts and titles, 43 full text studies were evaluated. Thirty-five articles were excluded as the full text was unavailable, or were conference abstracts, reviews and clinical trials lacking outcome data. Eight articles were finally selected, all of which were RCTs (Fig. 1).

### 3.2 Characteristics of the included studies

All the included trials were double-blind, placebo-controlled RCTs involving a total of 3788 patients with moderate to severe eosinophilic asthma. Patient age ranged from 12 to 75 years, and included 1573 males and 2215 females. Of these 3788 patients, 2277 received benralizumab treatment and the remaining 1511 received placebo, and a comparative analysis of different dose groups was included. All patients enrolled in the trials were from various experimental centers in a number of countries. All patients had a history of physician-diagnosed asthma requiring treatment with medium-to-high dose ICS or ICS/LABA for at least 1 year or 2 years prior to screening, and post-bronchodilator reversibility of airflow obstruction ≥12%. All female patients required adequate contraception from screening until trial completion. The patients in these trials experienced an exacerbation of asthma for at least 7 days or 12 months. The benralizumab group and placebo group were randomized to receive IV or SC injections as add-on to their standard background treatment. In addition to the patients in the study by Ferguson 2017 (in this study, patients receiving benralizumab and placebo gradually reduced the dose of oral glucocorticoid to a maintenance dose),^[18]^ patients in other studies were given benralizumab as an add-on treatment, and continued to receive their background asthma controller treatments (ICS or ICS/LABA) at a stable dosage during the study. All AEs were assessed up to day 1 after the last day of the study. Table 1 shows the characteristics of the included trials.

**Table 1:**
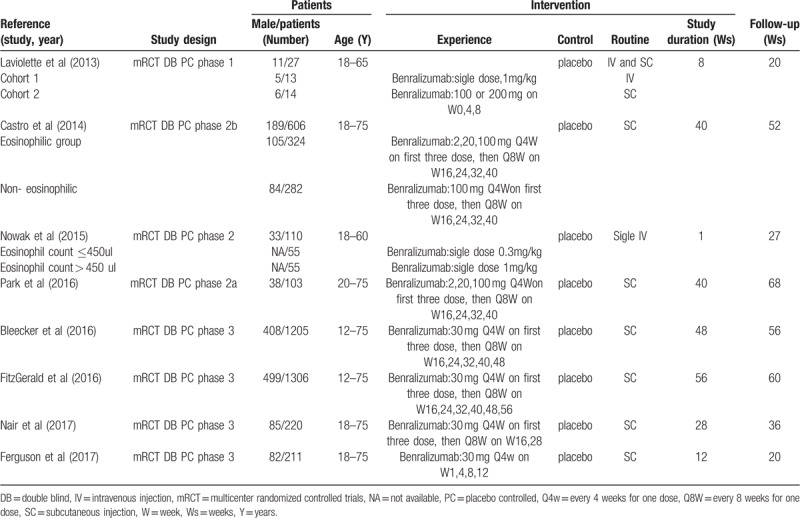
Characteristics of trials included in this study.

### 3.3 Risk of bias in the included studies

**Figure 2:**
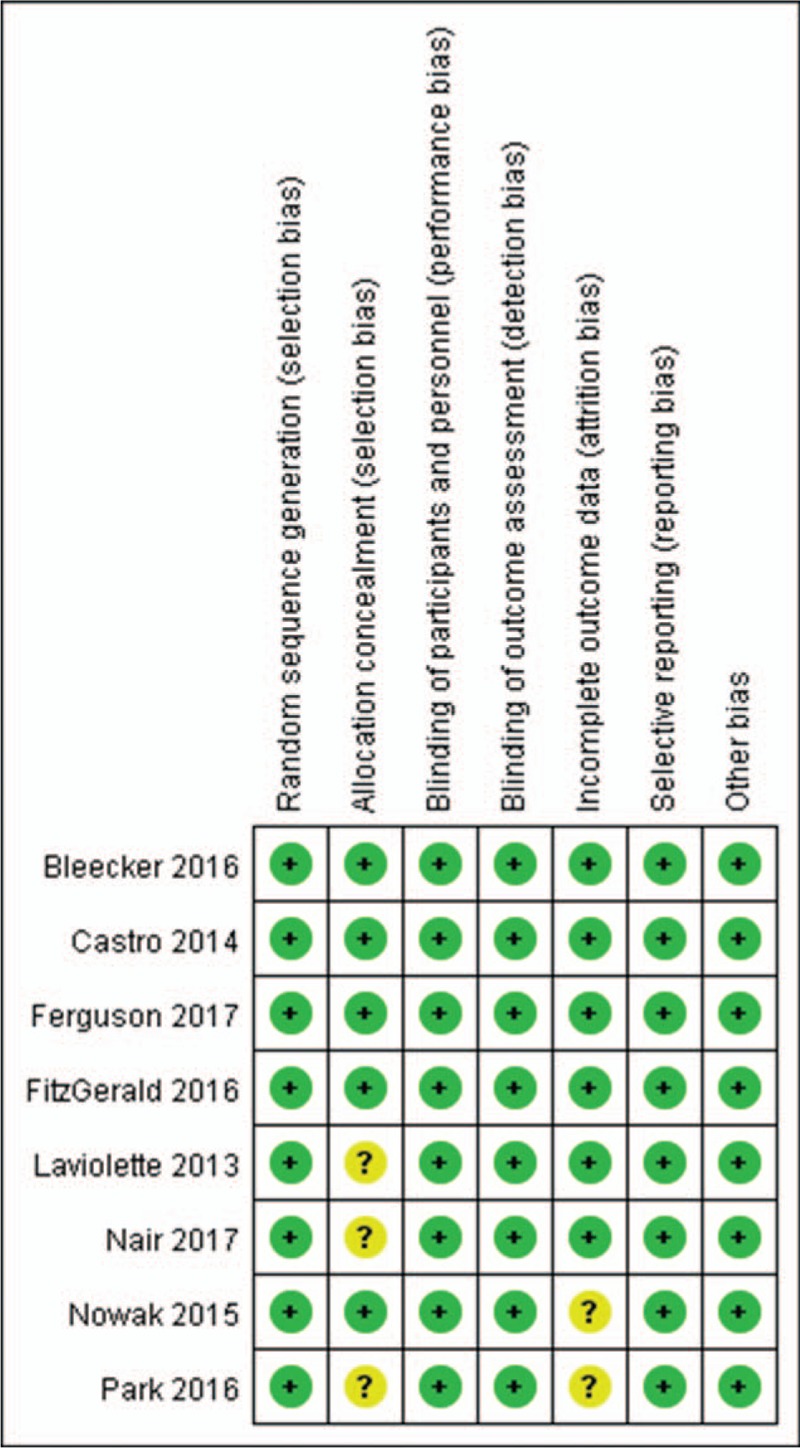
Risk-of-bias summary.

All the included trials were randomized trials with an interactive voice response system; thus, they were classified as having a low risk of selection bias. Only 3 trials did not describe the allocation concealment strategy^[11,16,17]^; thus, these trials were classified as having an unclear risk of bias. Allocation concealment was clear in the remaining trials. Blinding of participants was considered adequate in all studies; thus, blinding of participants and personnel, and blinding of outcome assessment in all studies included in this analysis were classified as having a low risk of selection bias. Blinding of outcome assessor was not mentioned in two trials^[13,16]^; thus, we classified these two trials as unclear risk. In all included trials, the loss to follow-up was less than 10%; therefore, all trials were classified as having a low risk of attrition bias. On account of all the outcomes specified in the methods reported, all trials were classified as having a low risk of reporting bias. The results of each risk-of-bias item for the included studies are summarized in Figure 2.

#### 3.3.1 Overall AEs

**Figure 3:**
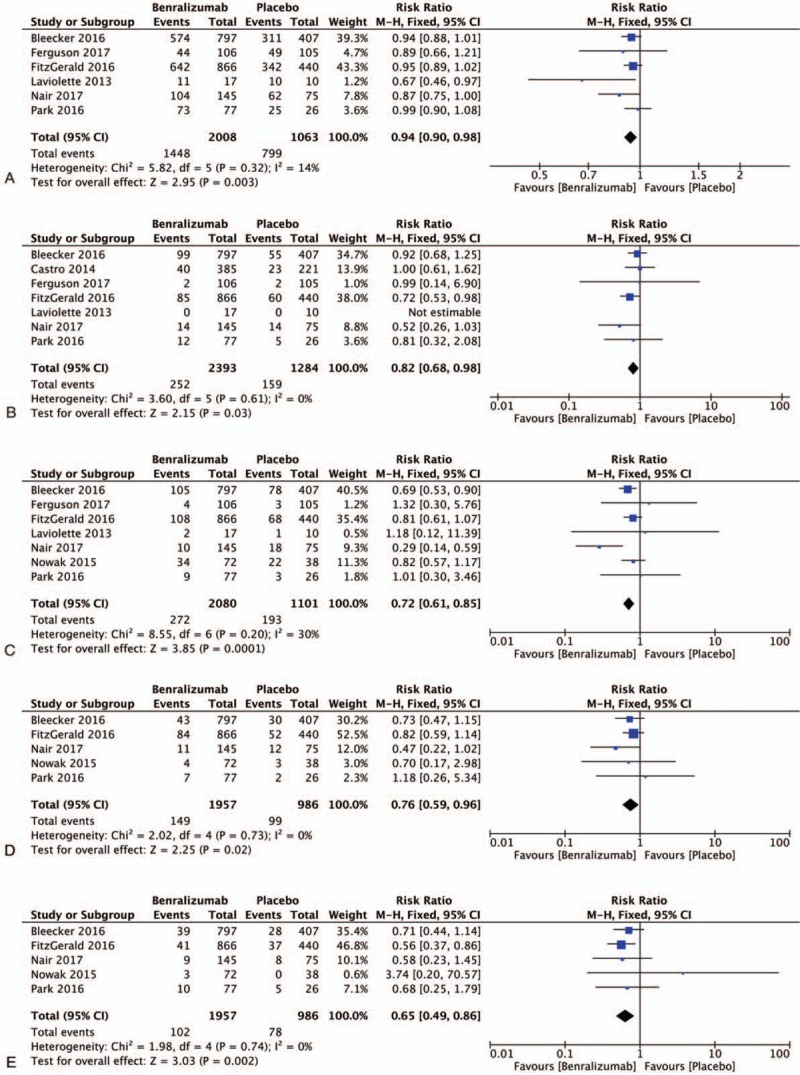
Meta-analysis of studies of the benralizumab group had a lower probability of adverse events than the placebo group; a, Overall AEs; b, SAEs; c, asthma; d, bronchitis; e, sinusitis; AEs: adverse events; SAEs: serious adverse events; H-X, Fixed, fixed effect model; CI, confidence interval.

Six trials^[13–17]^ reported overall AEs in this analysis: 7.61% (1448/2008) of patients in the benralizumab group developed bronchitis as compared to 10.04% (799/1063) of patients in the placebo group. Fewer patients treated with benralizumab vs placebo experienced overall AEs (RR 0.94, 95% CI 0.90–0.98, *P* = .03, I^2^ = 14%; Fig. 3a).

#### 3.3.2 Serious AEs (SAEs)

Few SAEs were caused by benralizumab in the trials included in this work. A total of 7 trials^[11,12,14–18]^ involving 3677 patients were included in this analysis, and one trial was not included as the data could not be extracted. SAEs were reported in 10.53% (252/2393) of patients treated with benralizumab and in 12.38% (159/1284) of patients treated with placebo. In the pooled analysis, patients receiving benralizumab had a lower chance of suffering SAEs than patients in the placebo group (RR 0.82, 95% CI 0.68–0.98, *P* = .03, I^2^ = 0%; Fig. 3b).

#### 3.3.3 Asthma

Worsening of asthma is one of the most frequently reported AEs. We pooled the data on worsening asthma, and a total of 7 trials^[11,13–18]^ were included. The benralizumab group included 2080 patients, and the placebo group included 1001 patients. The incidence of worsening asthma in the benralizumab group was 13.1% (272/2080), and the incidence in the placebo group was 17.53% (193/1101). A statistically significant difference in worsening asthma between the patients receiving benralizumab and patients receiving placebo was observed (RR 0.72, 95% CI 0.61–0.85, *P* = .0001, I^2^ = 30%; Fig. 3c). Thus, the benralizumab group had a lower risk of worsening asthma than the placebo group.

#### 3.3.4 Bronchitis

Five trials^[13–17]^ reported bronchitis in this analysis. The 7.61% (149/1957) of patients in the benralizumab group developed bronchitis as compared to 10.04% (99/986) of patients in the placebo group. The benralizumab group had a lower probability of bronchitis than the placebo group (RR 0.76, 95% CI 0.59–0.96, *P* = .02, I^2^ = 0%; Fig. 3d).

#### 3.3.5 Sinusitis

In the pooled analysis, 5.21% (102/1957) of patients receiving benralizumab experienced sinusitis as compared to 7.91% (78/986) of patients receiving placebo in 5 trials.^[13–17]^ Statistical analysis showed that the benralizumab group had a lower probability of experiencing sinusitis than the placebo group (RR 0.65, 95% CI 0.49–0.86, *P* = .002, I^2^ = 0%; Fig. 3e).

#### 3.3.6 Headache

**Figure 4:**
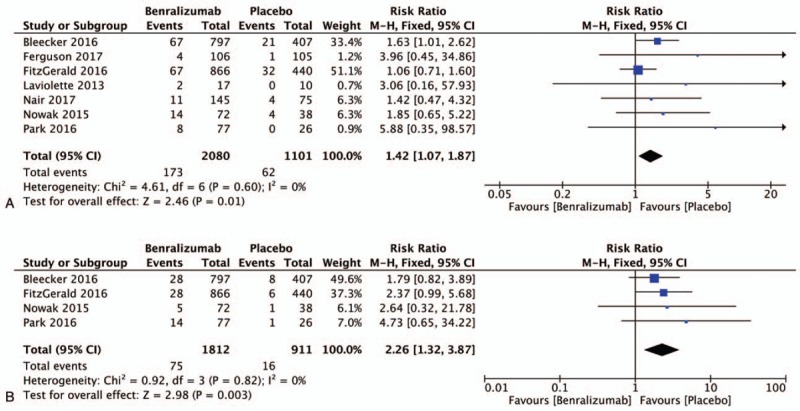
Meta-analysis of studies of the benralizumab group had a higher probability of adverse events than the placebo group; a, headache; b, pyrexia; H-X, Fixed, fixed effect model. CI, confidence interval.

Seven trials^[11,13–18]^ reported the incidence of headache: 8.32% (173/2080) of patients receiving benralizumab developed headache compared to 5.63% (62/1101) of patients receiving placebo. The benralizumab group was more likely to suffer headache than the placebo group (RR 1.42, 95% CI 1.07–1.87, *P* = .01, I^2^ = 0%; Fig. 4a).

#### 3.3.7 Pyrexia

Pyrexia was reported in 4 trials^[13–16]^ included in this analysis: 4.14% (75/1812) of patients receiving benralizumab developed pyrexia as compared to 1.76% (16/911) of patients receiving placebo. In these 4 trials, we found a higher odds of pyrexia in the benralizumab group as compared to the placebo group (RR 2.26, 95% CI 1.32–3.87, *P* = .003, I^2^ = 0%; Fig. 4b).

### 3.4 Other AEs

**Figure 5:**
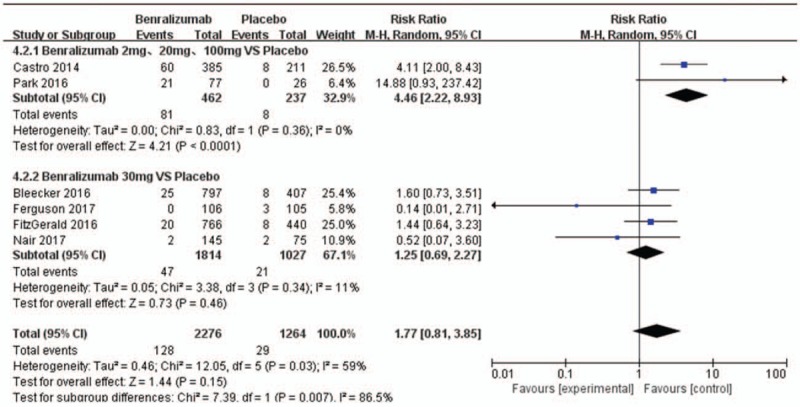
Meta-analysis of studies of injection-site reactions in patients treated with benralizumab or placebo; H-X, Fixed, fixed effect model. CI, confidence interval.

Six trials^[11,12,14–18]^ included in this analysis reported injection-site reactions. Results of the random effect analysis are presented as the heterogeneity was significant (I^2^ = 59%) in the subgroup analysis. We pooled the data on injection-site reactions and performed a subgroup analysis according to the dose of benralizumab received by the patients. The first subgroup included the patients in the study by Castro^[12]^ and Park^[16]^ based on mixed doses of benralizumab. Patients treated with 2 mg, 20 mg, or 100 mg benralizumab were included in the mixed dose group. The first subgroup displayed a higher probability of injection-site reactions in the mixed dose benralizumab group than in the placebo group (17.53% (81/462) and 3.38% (8/237), respectively; RR 4.46, 95% CI 2.22–8.93, *P* < .0001; Fig. 5). The studies by Bleecker,^[15]^ FitzGerald,^[14]^ Ferguson,^[18]^ and Nair^[17]^ were included in the second subgroup as all patients were treated with benralizumab at the dose of 30 mg. No significant difference in the probability of injection-site reactions was observed between the 30 mg benralizumab group and the placebo group (2.60% (47/1814) and 2.04% (21/1027), respectively; RR 1.25, 95% CI 0.69–2.27, *P* = .46; Fig. 5). The odds of injection-site reactions were comparable in the benralizumab group and the placebo group in the combined results for the 6 studies (1.27% (128/2276) and 2.36% (29/1231), respectively; RR 1.77, 95% CI 0.81–3.85, *P* = .15; Fig. 5). The remaining AEs associated with benralizumab were also analyzed in this study, such as death (see Data and Charts, Supplemental Content 1), hypersensitivity (see Data and Charts, Supplemental Content 2), nasopharyngitis (see Data and Charts, Supplemental Content 3), rhinitis (see Data and Charts, Supplemental Content 4), pharyngitis (see Data and Charts, Supplemental Content 5), upper respiratory tract infection (see Data and Charts, Supplemental Content 6), influenza (see Data and Charts, Supplemental Content 7), nausea (see Data and Charts, Supplemental Content 8), cough (see Data and Charts, Supplemental Content 9), back pain (see Data and Charts, Supplemental Content 10), and arthralgia (see Data and Charts, Supplemental Content 11). There were no significant differences in the incidence of these AEs between the benralizumab group and the placebo group (see Data and Charts, Supplemental Content 1–11, which illustrates that the incidence of the remaining AEs in the benralizumab group and placebo group was comparable).

## 4 Discussion

It is difficult to control persistent, moderate to severe eosinophilic asthma and reach a steady state with traditional ICS or ICS/LABA,^[21]^ and more effective therapies without severe AEs are required. The latest GINA guidelines advise the addition of anti-IL-5 treatment or IL-5 receptor treatment (subcutaneous benralizumab in patients aged ≥12 years) for severe eosinophilic asthma in the absence of a curative effect with ICS or ICS/LABA. Due to favorable efficacy and fewer AEs, benralizumab has been approved by the FDA as add-on maintenance therapy to treat severe asthma patients (≥12 years) with an eosinophilic phenotype.^[22]^ Although the efficacy and safety of benralizumab have been demonstrated, to date, there is some controversy regarding its AEs. There are few meta-analyses on the safety of benralizumab and these provided only a basic statistical analysis of the overall incidence of AEs and SAEs, and the types of AEs were not classified and analyzed. The present meta-analysis is the first to classify the most important and frequently observed AEs (SAEs, asthma exacerbation, bronchitis, sinusitis, headache, pyrexia, nasopharyngitis and death) in multiple trials and analyze the differences in these AEs between the benralizumab and placebo groups. It was found that some of these results were contrary to those observed in previous research.

Laviolette,^[11]^ Nowak,^[13]^ Bleecker,^[15]^ Nair,^[17]^ Ferguson,^[18]^ Tian,^[23]^ Liu,^[24]^ and Wang^[25]^ showed a comparable overall incidence of AEs in patients in the benralizumab and placebo groups during the study period. In addition, Castro^[12]^ and Park^[16]^ demonstrated that slightly more patients treated with benralizumab versus placebo experienced AEs. However, our results agree with those of FitzGerald^[14]^ that patients treated with benralizumab had a slightly lower risk of overall AEs than those treated with placebo (*P* = .003, I^2^ = 14%). It is worth mentioning that SAEs either did not occur or only a few occurred in previous trials of benralizumab, but these SAEs were still important during treatment. Henriksen^[26]^ analyzed nine studies involving 3193 patients and found a reduced risk of SAEs in the mepolizumab (SC) and reslizumab group (IV) groups compared to the placebo group. Farne^[27]^ found that significantly fewer SAEs occurred in the mepolizumab group (SC and IV), but there was no significant difference in the number of SAEs between that group and the benralizumab (SC) group. However, our meta-analysis found that patients treated with benralizumab had a lower chance of suffering SAEs than those treated with placebo (*P* = .03, I^2^ = 0%). The difference between the analysis by Farne^[27]^ and our analysis is that Farne^[27]^ only included patients treated with benralizumab (SC), and the subjects included in our study were treated with benralizumab (SC and IV). The patients receiving benralizumab IV had more asthma-related SAEs such as exacerbations requiring admission. Therefore, we believe that benralizumab can directly and rapidly deplete eosinophils and effectively reduce airway inflammation, thus alleviating the symptoms and signs of SAEs. Castro,^[12]^ Nowak,^[13]^ FitzGerald,^[14]^ and Bleecker^[15]^ showed that the most common SAE was worsening asthma^[14–17]^ and some SAEs (such as herpes zoster, polyarteritis nodosa, and uterine leiomyoma (Table 2) were closely related to the use of benralizumab. However, further investigations to verify these findings are required. It was believed that none of the deaths in these studies were related to treatment, which was comparable to our findings (*P* = .78, I^2^ = 0%; see Data and Charts, Supplemental Content). In summary, benralizumab, mepolizumab and reslizumab are all registered anti-IL-5 treatments for severe eosinophilic asthma. From the current study, these agents are safe, and can significantly alleviate symptoms in patients with moderate to severe asthma, and reduce the incidence of SAEs due to increased asthma.

**Table 2:**
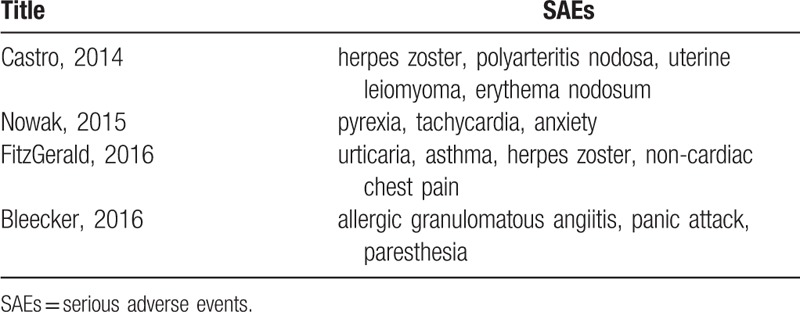
Serious adverse events related to benralizumab.

We also observed that benralizumab reduced the frequency of the moderate to severe eosinophilic asthma phenotype (*P* = .0001, I^2^ = 30%),^[11,13–18]^ bronchitis (*P* = .02, I^2^ = 0%) ^[13–17]^ and sinusitis (*P* = .002, I^2^ = 0%) ^[13–17]^ compared with the placebo group. This was consistent with our expectation. Eosinophils, stimulated by IL-5,^[28]^ can cause inflammation and the release of histamine.^[29]^ Platelet-activating factor secreted by eosinophils increases mucosal secretion of leukotriene C4, oxygen free radicals, degranulation of eosinophils,^[30]^ and aggravates asthma exacerbations, inflammation and local edema of nasal mucosa, and causes a decline in lung function. Benralizumab can directly, rapidly, and almost completely remove eosinophils via enhanced antibody-dependent cell-mediated cytotoxicity.^[18]^ Therefore, benralizumab improves the typical symptoms and signs in the eosinophilic asthma phenotype, reduces the rate of deterioration, increases FEV1, and alleviates the inflammation and symptoms of bronchitis and sinusitis. The above results indicate that benralizumab has a better additive effect on bronchitis in asthmatic patients. It is worth noting that there is no biological therapy for sinusitis approved by the FDA at present,^[31]^ and the data from this meta-analysis may provide vital information for the treatment of sinusitis. However, further investigation is required.

The center for drug evaluation and research of the FDA consider that headache and pharyngitis are the most common AEs in patients receiving benralizumab.^[22]^ Castro,^[12]^ FitzGerald,^[14]^ Park,^[16]^ Bleecker,^[15]^ Nair,^[17]^ and Ferguson^[18]^ reported that nasopharyngitis was the most common adverse event in the benralizumab and placebo groups. However, Castro^[12]^ demonstrated that the occurrence of nasopharyngitis was more than 5% higher in the benralizumab groups than in the placebo groups. It is worth mentioning that we found that the risk of headache was higher in the benralizumab group than in the placebo group (*P* = .01, I^2^ = 0%),^[11,13–18]^ while the incidence of pharyngitis was comparable between the 2 groups.^[11–18]^ (see Data and Charts, Supplemental Content). This is a new discovery and deserves further research. Patients treated with benralizumab were more likely to experience pyrexia than patients receiving placebo. We suspect that this may be related to the pharmacokinetics, and the specific mechanism is not detailed here.

The incidence of injection-site reactions did not differ significantly between the benralizumab group and the placebo group.^[11,12,14–18]^ However, heterogeneity (I^2^ = 59%) was observed in the entire group analysis of injection-site reactions, but in the subgroup analysis, in patients receiving different doses (2, 20, 100 mg) of benralizumab vs placebo and 30 mg benralizumab vs placebo, there was 0% and 11% heterogeneity (I^2^), respectively. The subgroup analysis showed that there was an increased risk of injection-site reactions in the different dose groups (2, 20, 100 mg) compared with the placebo group when the 2 effects models were changed. However, the data on patients who received 2 mg, 20 mg, and 100 mg of benralizumab could not be separately extracted, and these results require further investigation to support these findings. There was no significant difference in injection-site reactions between the 30 mg benralizumab group and placebo group when the 2 effects models were changed. Therefore, we consider that the heterogeneity observed in the entire group analysis of injection-site reactions was closely related to the dose of benralizumab received.

The incidence of other AEs, such as death, hypersensitivity, rhinitis, upper respiratory tract infection, influenza, nausea, cough, back pain, and arthralgia was comparable in patients treated with benralizumab and placebo (see Data and Charts, Supplemental Content, which illustrates that the incidence of other AEs between the benralizumab group and placebo group was comparable).

Meta-analysis plays an important role in assessing relevant disputes. As far as we know, this article is the first meta-analysis to classify and analyze the AEs of benralizumab. This meta-analysis compared the AEs of benralizumab, mepolizumab and reslizumab, and found that all three are safe and can reduce SAEs during the treatment of asthma patients. This study provides doctors with an essential reference on the efficacy and safety of benralizumab in clinical treatment. The advantage of this meta-analysis is that all the studies were placebo-controlled RCTs. Another strength was the low heterogeneity in the majority of outcomes among the studies.

Our study had several limitations. Firstly, the data on specific doses (2, 20,100 mg) in patients treated with benralizumab were not extractable in the subgroup analysis of injection-site reactions. Secondly, this meta-analysis included only eight clinical trials that were all funded by the pharmaceutical industry. Furthermore, the possibility of publication bias is an ever-present limitation of the statistical method in the meta-analysis. Nevertheless, it was difficult to assess publication bias due to the small number of studies included in the meta-analysis.

## 5 Conclusion

This meta-analysis is the first study to classify and comprehensively assess the risk of AEs caused by benralizumab. Like mepolizumab and reslizumab, benralizumab reduces the risk of SAEs and significantly alleviates the symptoms of moderate to severe asthma. In addition, benralizumab can reduce asthma exacerbations, and the incidence of bronchitis, and sinusitis. Furthermore, we found that headache, and pyrexia were more likely to occur in patients treated with benralizumab than with placebo. There was no difference in the likelihood of certain AEs occurring in patients receiving benralizumab compared with those receiving placebo, including the most common AEs reported in a number of trials nasopharyngitis, death, hypersensitivity, injection-site reactions, rhinitis, pharyngitis, upper respiratory tract infection, influenza, nausea, cough, back pain, and arthralgia. In conclusion, benralizumab as an add-on treatment has commendable efficacy and safety in patients with moderate to severe eosinophilic asthma with little benefit on ICS or ICS/LABA treatment. However, vigilance to assess the occurrence of AEs in any patient receiving immunomodulatory therapy must be maintained. The long-term safety and effectiveness of benralizumab require further research.

## Author contributions

Wanshu Liu and Xuesu Ma contributed equally to the work as co-first authors. Wanshu Liu and Xuesu designed the study, and wrote the manuscript; Wanshu Liu, Xingru Wu and Zuzhen Ou analyzed the data. Weikang Zhou edited the manuscript. In addition, all authors approved the final draft.

**Formal analysis:** Wanshu Liu.

**Methodology:** Weikang Zhou.

**Writing – original draft:** Wanshu Liu, Xuesu Ma.

**Writing – review & editing:** Weikang Zhou.

Wanshu Liu orcid: 0000-0003-0848-1147.

## Supplementary Material

Supplemental Digital Content
