# Supplementary material for: Adverse events of benralizumab in moderate to severe eosinophilic asthma: A meta-analysis
Source: Medicine (Baltimore). 2019 May 31;98(22):e15868. doi: 10.1097/MD.0000000000015868 (PMC6709166; doi:10.1097/MD.0000000000015868)
Supplement: Supplemental Digital Content [file medi-98-e15868-s001.pdf]

Supplemental Content 1

Title: Adverse events of Benralizumab in moderate-to-severe eosinophilic asthma: A meta-analysis

Co-first authors: Wanshu Liu, Xuesu Ma

Supplemental content 1: The data and charts of death

In 8 trials, death was reported in 0.45% (11/2465) of patients treated with benralizumab and in 0.23% (3/1322) of patients treated with a placebo. No significant difference was noted between the benralizumab group and placebo group (RR 1.68, 95%CI 0.55—5.14, P=0.37, I^2=0%; Figure 1s).

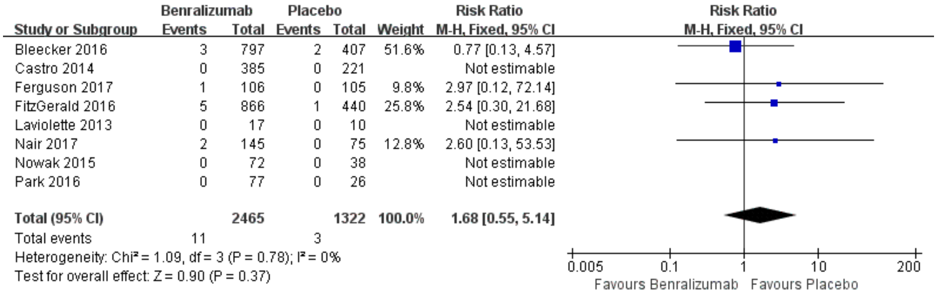

Figure 1s. Meta-analysis of studies of the probability of the death was similar between the benralizumab group and placebo group; H-X, Fixed, fixed effect model. CI, confidence interval.

Supplemental Content 2

Title: Adverse events of Benralizumab in moderate-to-severe eosinophilic asthma: A meta-analysis

Co-first authors: Wanshu Liu, Xuesu Ma

Supplemental content 2: The data and charts of Hypersensitivity.

In 4 trials, hypersensitivity was reported in 2.87% (55/1914) of patients treated with benralizumab and in 2.92% (30/1027) of patients treated with placebo. There were no statistically significant differences between the benralizumab group and placebo group (RR 0.96, 95%CI 0.62—1.48, P=0.85, I^2=0%; Figure 2s).

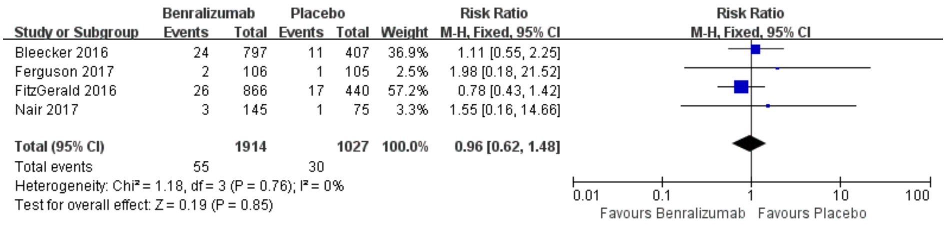

Figure 2s. Meta-analysis of studies of the probability of the hypersensitivity was similar between the benralizumab group and placebo group; H-X, Fixed, fixed effect model. CI, confidence interval.

Supplemental Content 3

Title: Adverse events of Benralizumab in moderate-to-severe eosinophilic asthma: A meta-analysis

Co-first authors: Wanshu Liu, Xuesu Ma

Supplemental content 3: The data and charts of nasopharyngitis.

Nasopharyngitis was one of the most frequently reported AEs in all the trials included in this analysis. The nasopharyngitis data revealed that 15.36% (365/2377) of patients receiving benralizumab developed nasopharyngitis, and 14.67% (194/1322) of patients receiving placebo developed nasopharyngitis. There was no significant difference in the incidence of nasopharyngitis between the benralizumab group and the placebo group (RR 1.03, 95%CI 0.88—1.21, P=0.71, I^2=40%; Figure 3s).

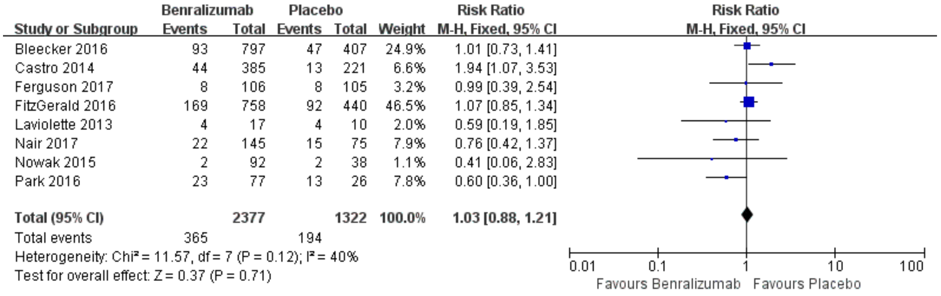

Figure 3s. Meta-analysis of studies of the probability of the nasopharyngitis was similar between the benralizumab group and placebo group; H-X, Fixed, fixed effect model. CI, confidence interval.

Supplemental Content　4

Title: Adverse events of Benralizumab in moderate-to-severe eosinophilic asthma: A meta-analysis

Co-first authors: Wanshu Liu, Xuesu Ma

Supplemental content 4: The data and charts of Rhinitis.

Four trials included in this analysis reported rhinitis. 3.55% (67/1885) of patients receiving benralizumab developed rhinitis as compared to 2.68% (34/948) of patients receiving placebo. No statistically significant difference in the incidence of rhinitis was observed between the benralizumab group and placebo group (RR 0.98, 95%CI 0.65—1.47, P=0.92, I²=0%; Figure 4s).

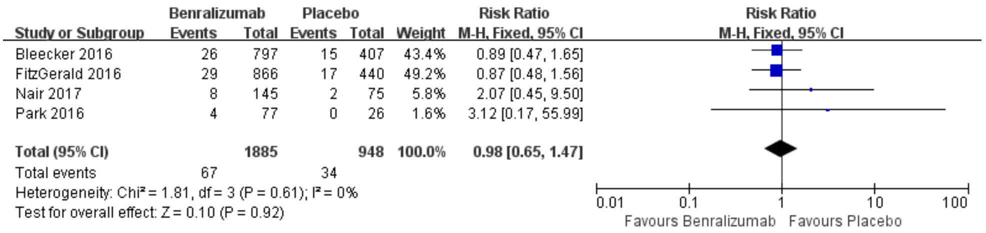

Figure 4s. Meta-analysis of studies of the probability of the rhinitis was similar between the benralizumab group and placebo group; H-X, Fixed, fixed effect model. CI, confidence interval.

Supplemental Content 5

Title: Adverse events of Benralizumab in moderate-to-severe eosinophilic asthma: A meta-analysis

Co-first authors: Wanshu Liu, Xuesu Ma

Supplemental content 5: The data and charts of Pharyngitis.

Three trials included in this analysis reported the incidence of pharyngitis. 3.33% (58/1740) of patients in the benralizumab group developed pharyngitis compared with 2.75% (24/873)of patients in the placebo group. No statistically significant difference was observed between the benralizumab group and the placebo group (RR 1.19, 95%CI 0.74—1.90, P=0.47, I²=5%; Figure 5s).

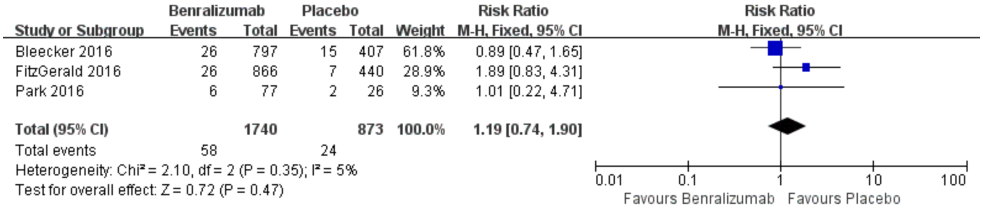

Figure 5s. Meta-analysis of studies of the probability of the pharyngitis was similar between the benralizumab group and placebo group; H-X, Fixed, fixed effect model. CI, confidence interval.

Supplemental Content 6

Title: Adverse events of Benralizumab in moderate-to-severe eosinophilic asthma: A meta-analysis

Co-first authors: Wanshu Liu, Xuesu Ma

Supplemental content 6: The data and charts of Upper respiratory tract infection.

Six trials included in this analysis reported upper respiratory tract infection. 8.58% (177/2063) of patients in the benralizumab group developed upper respiratory tract infection as compared to 8.98% (98/1091) of patients in the placebo group. No statistically significant difference was observed between the two groups (RR 0.91 95%CI 0.72—1.15, P=0.42, I^2=0%; Figure 6s).

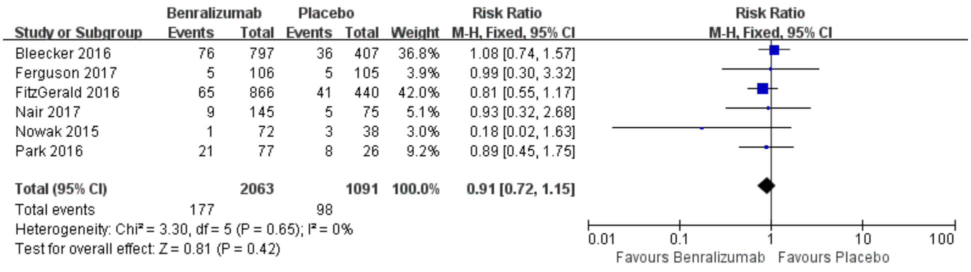

Figure 6s. Meta-analysis of studies of the probability of the upper respiratory tract infection was similar between the benralizumab group and placebo group; H-X, Fixed, fixed effect model. CI, confidence interval.

Supplemental Content　7

Title: Adverse events of Benralizumab in moderate-to-severe eosinophilic asthma: A meta-analysis

Co-first authors: Wanshu Liu, Xuesu Ma

Supplemental content 7: The data and charts of Influenza

In these trials, 4.62% (87/1885) of patients receiving benralizumab experienced influenza as compared to 5.60% (53/948) of patients receiving placebo. No statistically significant difference was observed between the benralizumab group and the placebo group (RR 0.81,95%CI 0.58—1.13, P=0.21, I²=10%; Figure 7s).

	Benralizumab		Placebo			Risk Ratio
Study or Subgroup	Events	Total	Events	Total	Weight	M-H, Fixed, 95% CI
Bleecker 2016	36	797	23	407	43.3%	0.80 [0.48, 1.33]
FitzGerald 2016	36	866	24	440	45.2%	0.76 [0.46, 1.26]
Nair 2017	4	145	5	75	9.4%	0.41 [0.11, 1.50]
Park 2016	11	77	1	26	2.1%	3.71 [0.50, 27.40]
**Total (95% CI)**		**1885**		**948**	**100.0%**	**0.81 [0.58, 1.13]**
Total events	87		53			
Heterogeneity: Chi² = 3.33, df = 3 (P = 0.34); I² = 10%						
Test for overall effect: Z = 1.24 (P = 0.21)						

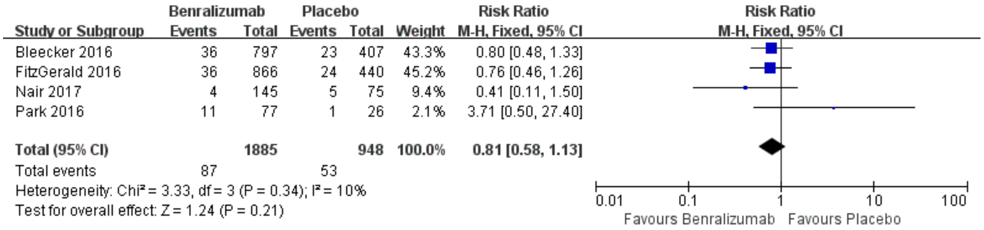

Figure 7s. Meta-analysis of studies of the probability of the influenza was similar between the benralizumab group and placebo group; H-X, Fixed, fixed effect model. CI, confidence interval.

Supplemental Content 8

Title: Adverse events of Benralizumab in moderate-to-severe eosinophilic asthma: A meta-analysis

Co-first authors: Wanshu Liu, Xuesu Ma

Supplemental content 8: The data and charts of Nausea.

Four trials included in this analysis reported nausea. 3.12% (8/251) of patients in the benralizumab group experienced nausea as compared to 4.51% (6/133) of patients in the placebo group. No statistically significant difference was observed between the benralizumab group and the placebo group (RR 1.04, 95%CI 0.54—2.00, P=0.91, I²=0%; Figure 8s).

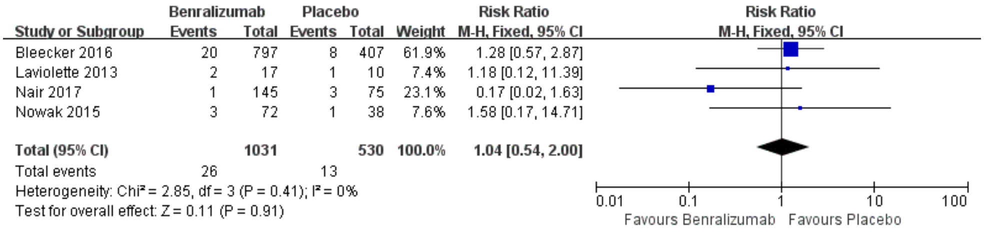

Figure 8s. Meta-analysis of studies of the probability of the nausea was similar between the benralizumab group and placebo group; H-X, Fixed, fixed effect model. CI, confidence interval.

Supplemental Content 9

Title: Adverse events of Benralizumab in moderate-to-severe eosinophilic asthma: A meta-analysis

Co-first authors: Wanshu Liu, Xuesu Ma

Supplemental content 9: The data and charts of cough.

Five trials included in this analysis reported cough. 3.32% (63/1897) of patients in the benralizumab group developed cough as compared to 2.68% (26/970) of patients in the placebo group. No statistically significant difference was observed between the benralizumab group and the placebo group (RR 1.25, 95%CI 0.79—1.95, P=0.34, I²=0%; Figure 9s).

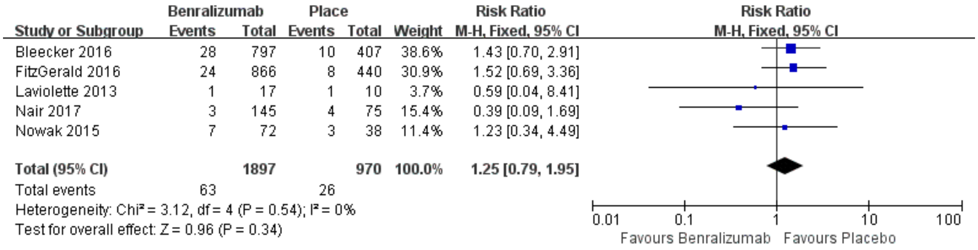

Figure 9s. Meta-analysis of studies of the probability of the cough was similar between the benralizumab group and placebo group; H-X, Fixed, fixed effect model. CI, confidence interval.

Supplemental Content 10

Title: Adverse events of Benralizumab in moderate-to-severe eosinophilic asthma: A meta-analysis

Co-first authors: Wanshu Liu, Xuesu Ma

Supplemental content 10: The data and charts of back pain.

Five trials included in this analysis reported back pain. 2.86% (56/1957) of patients treated with benralizumab developed back pain as compared to 3.85% (38/986) of patients treated with placebo. No statistically significant difference was observed between the benralizumab group and the placebo group (RR 0.73, 95%CI 0.49—1.10, P=0.13, I^2=0%; Figure 10s).

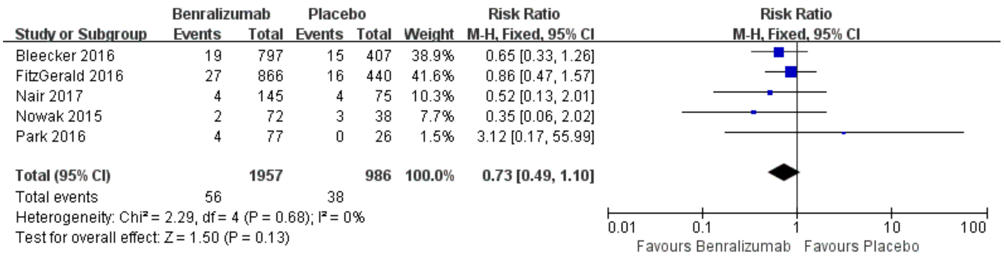

Figure 10s. Meta-analysis of studies of the probability of the back pain was similar between the benralizumab group and placebo group; H-X, Fixed, fixed effect model. CI, confidence interval.

Supplemental Content 11

Title: Adverse events of Benralizumab in moderate-to-severe eosinophilic asthma: A meta-analysis

Co-first authors: Wanshu Liu, Xuesu Ma

Supplemental content 11: The data and charts of Arthralgia.

Arthralgia

Four trials included in this analysis reported arthralgia.3.20% (58/1812) of patients treated with benralizumab developed arthralgia as compared to 2.41% (22/911) of patients treated with placebo. No statistically significant difference was observed between patients receiving benralizumab and those receiving placebo (RR 1.30, 95%CI 0.80—2.12, P=0.29, I²=0%; Figure 11s)

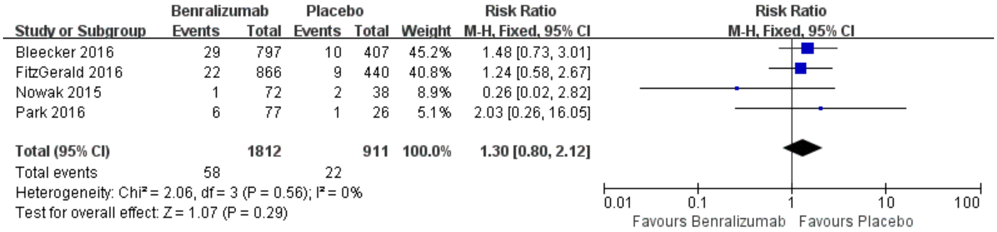

Figure 11s. Meta-analysis of studies of the probability of the arthralgia was similar between the benralizumab group and placebo group; H-X, Fixed, fixed effect model. CI, confidence interval.
